# Changes in Underweight Status and Risk of Hip Fracture: A Korean Nationwide Population-Based Cohort Study

**DOI:** 10.3390/jcm11071913

**Published:** 2022-03-30

**Authors:** Sangsoo Han, Jiwon Park, Hae-Dong Jang, Kyungdo Han, Choungah Lee, Wonseok Kim, Jae-Young Hong

**Affiliations:** 1 Department of Emergency Medicine, Soonchunhyang University Bucheon Hospital, 170 Jomaru-ro, Bucheon 14584, Korea; brayden0819@schmc.ac.kr; 2 Department of Orthopedics, Korea University Ansan Hospital, 123, Jeokgeum-ro, Danwon-gu, Ansan-si 15355, Korea; jwpark506@gmail.com (J.P.); znzooof@naver.com (W.K.); 3 Department of Orthopaedic Surgery, Soonchunhyang University Bucheon Hospital, 170 Jomaru-ro, Bucheon 14584, Korea; khaki00@schmc.ac.kr; 4 Department of Statistics and Actuarial Science, Soongsil University, 369 Sangdo-ro, Dongjak-gu, Seoul 06978, Korea; hkd917@naver.com; 5 Department of Emergency Medicine, Hallym University Dongtan Sacred Heart Hospital, 7 Keunjaebong-gil, Hwaseong-si 18450, Korea; cuccum@hanmail.net

**Keywords:** cohort study, hip fracture, risk factor, underweight

## Abstract

Being underweight is associated with a high risk of hip fracture. However, the impact of change in underweight status on the risk of hip fracture is unknown. This study is performed to investigate the relationship between change in underweight status and risk of hip fracture. This study included 1,713,225 subjects aged ≥40 years who underwent two consecutive national health screenings between 2007 and 2009. We prospectively assessed the risk of hip fracture between 2010 and 2018 according to changes in underweight status. We divided the participants into four groups according to the change in underweight status: consistent non-underweight (non-underweight to non-underweight), became non-underweight (underweight to non-underweight), became underweight (non-underweight to underweight), and consistent underweight (underweight to underweight). Compared with the consistent non-underweight group, the became non-underweight (0.74/1000 person years (PY) increase in incidence rate (IR); adjusted hazard ratio (HR) 1.72; 95% confidence interval (CI) 1.42–2.07), became underweight (1.71/1000 PY increase in IR; adjusted HR 2.22; 95% CI 1.96–2.53), and consistent underweight (1.3/1000 PY increase in IR; adjusted HR 2.18; 95% CI 1.89–2.53) groups had a significantly increased risk of hip fracture (*p* < 0.001). Change in underweight status was significantly associated with a risk of hip fracture.

## 1. Introduction

The incidence of hip fractures is increasing with the aging of society worldwide [1,2]. There were 1.6 million hip fractures annually worldwide in 2000, and this number is expected to triple by 2050 [3]. As hip fractures are associated with increased economic costs for both patients and society, they are considered a major public health concern [4,5]. On average, the life expectancy of those with hip fractures is reduced by 2.7%, and 12–58% of elderly people die within 1 year after hip fracture [6,7].

Being underweight is a well-known risk factor for hip fracture. A cohort study of 24,691 Japanese participants aged ≥75 years found that the risk of hip fracture in people who were underweight was up to 1.74 times higher than that in participants of normal weight [8]. In addition, in a meta-analysis involving 1,508,366 patients, being underweight was reported to be associated with a significantly increased risk of hip fracture, with a pooled odds ratio of 2.83 [9]. Recent studies have reported that intentional weight loss increases the risk of fracture [10,11]. However, the relationship between a change in underweight status and risk of hip fracture among adults remains unclear.

In this paper, we examine the associations of changes in underweight status with risk of hip fracture using large population-based data from adults over 40 years of age with records of sociodemographic factors, health surveys, tests, and medical claims from the National Health Insurance Service (NHIS) of Korea.

## 2. Materials and Methods

### 2.1. Data Collection and Study Population

This nationwide population-based observational cohort study was conducted using claims data from the NHIS in Korea. NHIS is a quasi-governmental institution under the Ministry of Health and Welfare that has provided compulsory medical insurance services covering almost 99% of the Korean population since 1989. NHIS provides health checkups once every 2 years for adults with national health insurance. The NHIS database contains information on demographics, national health screening (general health examination results and questionnaires on lifestyle and behavior), medical treatment, and comorbidities (diagnosis statement defined by the International Statistical Classification of Diseases and Related Health Problems [ICD-10]). All data are anonymized, collected regularly, and carefully quality controlled. Since 2015, NHIS has been providing data to researchers whose research protocols have been approved by the official review committee.

We enrolled participants of at least 40 years of age who underwent two consecutive biennial national health screenings provided by the NHIS between 2007 and 2009. The exclusion criteria were missing data, a prior fracture before the health check-up, and a 1-year lag period.

### 2.2. Measurements

We extracted demographic, socioeconomic, and clinical data from the NHIS database: sex, age, height, weight, smoking status (non-smoker, ex-smoker, or current smoker), alcohol consumption (non-drinker, moderate drinker (<30 g/day), or heavy drinker (≥30 g/day)), regular exercise (at least 20 min of vigorous physical activity ≥3 days per week or ≥30 min of moderate-intensity physical activity ≥5 days per week), low income (<20th percentile), blood glucose level, total cholesterol level, blood pressure, and estimated glomerular filtration rate.

The definitions of comorbidities based on ICD codes were taken from those validated in previous studies [12,13,14]. Diabetes was defined as a fasting glucose level >126 mg/dL or at least one claim per year under ICD-10 codes E10–14 with a prescription of an antidiabetic drug. Hypertension was defined as an average systolic/diastolic blood pressure ≥140/90 mmHg or at least one claim per year under ICD-10 codes I10–I13 or I15 with prescription of an antihypertensive drug. Dyslipidemia was defined as a total cholesterol level ≥240 mg/dL or at least one annual prescription of an antihyperlipidemic drug under ICD-10 code E78. Chronic kidney disease (CKD) was defined as estimated glomerular filtration rate <60 mL/min/1.73 m^2^.

### 2.3. Classification of Change in Underweight Status

Body mass index (BMI) was calculated by dividing body weight (kg) by height squared (m^2^). Underweight was defined as BMI < 18.5. We examined whether the participants were underweight by BMI measurements at two examinations (2007 and 2009), and then the study population was divided into four groups according to the change in underweight status: consistent non-underweight (CN, non-underweight to non-underweight), became non-underweight (BN, underweight to non-underweight), became underweight (BU, non-underweight to underweight), and consistent underweight (CU, underweight to underweight).

### 2.4. Definition of Study Outcome and Follow-Up Duration

Hip fracture was defined as one hospitalization under ICD-10 code S72.0, S72.1, or S72.2. We identified hip fracture events in the NHIS claims records from 1 January 2010 to 31 December 2018. Participants who died during the follow-up period were censored at the time of death.

### 2.5. Statistical Analysis

Baseline characteristics are presented as numbers with percentages for categorical variables and as numbers with standard deviations for continuous variables. The risk of hip fracture is presented as a hazard ratio (HR) with 95% confidence interval (CI) based on Cox regression analysis. The incidence rate (IR) was calculated as the number of hip fractures per 1000 person years (PY). Four proportional hazard models were constructed to investigate covariates potentially associated with hip fracture: model 1 was unadjusted; model 2 was adjusted for age and sex; model 3 was additionally adjusted for alcohol consumption, smoking status, regular exercise, and household income; and model 4 was further adjusted for comorbidities (diabetes, hypertension, dyslipidemia, and CKD). We compared the cumulative incidence of hip fractures between groups using the Kaplan–Meier method. We also conducted subgroup analyses based on age (<65 and ≥65 years) and sex. In all analyses, *p* < 0.05 (two-sided) was taken to indicate statistical significance. All statistical analyses were performed using SAS software (ver. 9.3; SAS Institute, Cary, NC, U.S.A.).

## 3. Results

**Figure 1:**
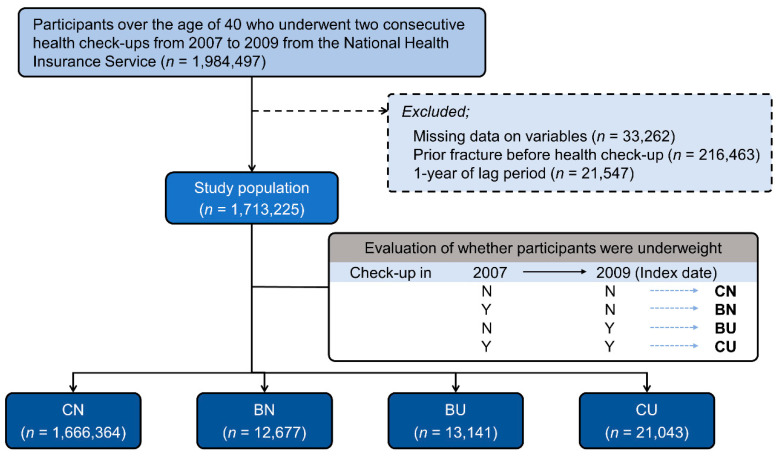
Flow chart of the present study. Y, yes; N, no; CN, consistent non-underweight; BN, became non-underweight; BU, became underweight; CU, consistent underweight.

A total of 1,984,497 adults over the age of 40 years who received consecutive health checkups provided by NHIS in 2007 and 2009 were initially included in the study. Among these participants, 33,262 with missing data, 216,463 with fractures before the medical examination, and 21,547 with fractures during the 1-year lag period were excluded. Therefore, 1,713,225 participants were included in the final analysis (Figure 1).

### 3.1. Baseline Characteristics

The 1,713,225 participants were divided into four groups according to the change in underweight status: CN group (1,666,364 participants), BN group (12,677 participants), BU group (13,141 participants), and CU group (21,043 participants). Comparisons of baseline characteristics among these groups using the change in underweight status are shown in Table 1. Due to the very large size of the study population, *p*-values for all variables were <0.001. The BU group had the oldest average age of 57.7 years, followed by the CU group (56.1 years), CN group (55.2 years), and BN group (55.1 years). The CN group had a higher proportion of males than females (52.6% vs. 47.4%, respectively), but there were fewer males than females in the BN (44.2% vs. 55.8%, respectively), BU (43.6% vs. 56.4%, respectively), and CU groups (56.9% vs. 53.1%, respectively). The incidence of hip fracture was the highest in the BU group (189 participants, 1.4%), followed by the CU group (252 participants, 1.2%), BN group (110 participants, 0.9%), and CN group (6885 participants, 0.41%).

### 3.2. Risk of Hip Fracture according to Change in Underweight Status

After adjustments for multiple variables (age, sex, smoking, alcohol consumption, household income, regular exercise, and comorbidities), the participants in the BU group (1.71/1000 PY increase in IR; HR 2.22; 95% CI 1.96–2.53), CU group (1.3/1000 PY increase in IR; HR 2.18; 95% CI 1.89–2.53), and BN group (0.74/1000 PY increase in IR; HR 1.72; 95% CI 1.42–2.07) had a significantly increased risk of hip fracture events compared with those in the CN group (Table 2).

**Figure 2:**
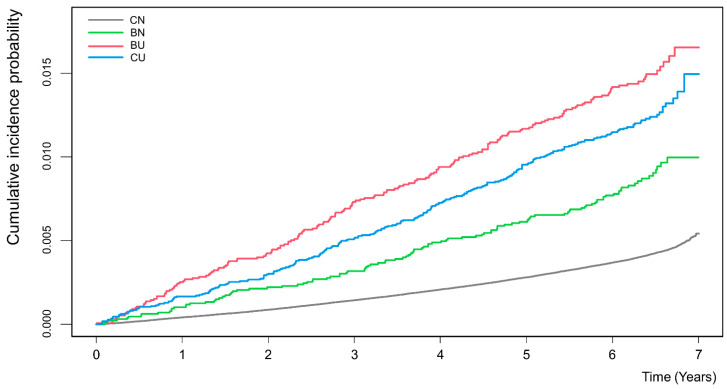
Estimates of cumulative incidence of hip fracture according to the change in underweight status. CN, consistent non-underweight; BN, became non-underweight; BU, became underweight; CU, consistent underweight.

The cumulative incidence was estimated using the Kaplan–Meier method in Figure 2. The BU group showed a significantly higher cumulative hip fracture incidence compared with the other groups at all time points, followed by the CU, BN, and CN groups.

### 3.3. Subgroup Analyses of Hip Fracture Risk

We performed subgroup analyses by stratifying the study population according to age and sex. The adjusted hip fracture risks according to the change in underweight status in each subgroup are presented in Table 3. In the subgroups according to age, adjusted HRs for the BN, BU and CU groups were 1.74, 3.13 and 3.08, respectively, in participants ≥65 years old, but were 1.7, 2.02 and 2.04, respectively, in those <65 years old (*p* = 0.012). In the subgroups according to sex, adjusted HRs for the BN, BU, and CU groups were 1.95, 2.84, and 2.83, respectively, in males, but 1.55, 1.75 and 1.73, respectively, in females (*p* < 0.001).

## 4. Discussion

Using a large national population-based database, we found real-world evidence that the change in underweight status was significantly correlated with hip fracture risk. In the data obtained through two consecutive health checkups, we confirmed that the BU group, in which the participants changed from non-underweight to underweight, had the highest risk of hip fracture. That is, even if the initial weight was normal, the risk of hip fracture can be rather high, if changed to become underweight at the re-examination after 2 years. Our results support that additional education on these risks may be beneficial even in normal weight patients. We also confirmed that the cumulative probability of hip fracture was the highest in the BU group at all time points.

Well-known risk factors for hip fracture include age, sex, height, weight, race, prior fracture, cognitive dysfunction, low socioeconomic status, lack of physical activity, and health behaviors, such as smoking and alcohol consumption [15,16]. We adjusted for the known risk factors as much as possible based on the information in the NHIS database to explore the relationship between the change in underweight status and risk of hip fracture.

In the present study, the BN group showed a 1.72 times greater hip fracture risk compared with the CN group. In general, when body weight is regained, body composition changes include more fat than muscle mass [17,18]. This can lead to the accumulation of unhealthy fat, which can further reduce bone mass and strength [19,20]. In addition, in our study, the BU and CU groups showed a significantly higher risk of hip fracture compared with the CN group. This can be explained as follows. First, greater soft tissue padding in the hip could counteract the traumatic forces [10]. Therefore, people who are underweight are more vulnerable to hip fracture because they have insufficient fat padding. Second, being underweight decreases the mechanical load on weight bearing, which may result in altered bone remodeling processes [21]. Third, underweight may be associated with a reduced intake of nutrients, including calcium and protein. Calcium deficiency can decrease bone mineral density (BMD), and protein depletion can reduce the production of insulin-like growth factor 1, affecting the bone remodeling process [22,23]. Finally, being underweight may be related to sarcopenia [24]. Reduced muscle mass may not provide adequate bone protection, and reduced muscle strength may increase the risk of fall-related injuries [25,26].

In our study, the association between a change in underweight status and hip fracture risk was greater in elderly subjects (≥65 years) than younger subjects (<65 years). Coin et al. reported that being underweight in the elderly was significantly associated with osteoporosis [27]. In addition, fall-related injuries, such as hip fractures, are more likely to occur in older adults due to conditions such as muscle weakness or impaired vision and balance [28].

In our study, the association between hip fracture risk and low body weight change was stronger in men than in women. According to a recent large-scale Norwegian cohort study, the risk of hip fracture in underweight individuals with a BMI ≤ 22 was higher in men than in women [29]. The sex-related differences in fat distribution are reflected by a relatively central fat distribution in men versus fat accumulation in the hip area in women [30]. Therefore, for women, the soft tissue padding in the hips provides protection during a fall.

A notable strength of this study is that we used a large sample from a nationally representative database. In addition, we analyzed data from two consecutive national health examinations to explore the association between a change in underweight status and the risk of hip fracture. Our results mean that, even if there is an underweight classification at one point in time, the risk is not the same, and may vary according to the change in BMI over two consecutive years. It also shows that, even if the patient is of normal weight, they may be at greater risk if they become underweight in the future. Through this, clinicians may be able to screen and monitor patients more precisely. To our knowledge, there have been no previous reports of a relationship between a change in underweight status and the risk of hip fracture.

However, this study had several limitations. First, there may have been selection bias due to the exclusion of subjects who did not undergo two consecutive health checkups. Although the NHIS provides mandatory national health insurance to all citizens in Korea, there is a possibility that people in certain socioeconomic status brackets may not be able to receive health checkups. Second, the incidence of hip fracture may be underestimated because the data from the NHIS can only identify patients who have received treatment in a hospital. Additionally, the change in BMI in each group may have been small. Third, caution is required in generalizing the results to other races. Race may also influence the relationship between BMI and fractures. BMD not only differs according to race, but has been reported to differ in the risk of fracture according to race even after excluding the effects of BMD [31,32]. Fourth, we could not follow up on the weight change over time because we analyzed the BMI of two consecutive checkup data. If the weight change was followed, the hip fracture incidence may have been affected by the change. Fifth, the NHIS database lacked data related to risk factors, such as BMD, steroid use, and family history of fracture, so we could not consider such factors. Low BMD, use of steroids, and parent history of fractured hip are known factors for future hip fracture, which are also used in the Fracture Risk Assessment Tool (FRAX) [33]. Sixth, the causal relationship between the risk of hip fracture and the change in underweight status could not be confirmed due to the study design. Therefore, further studies are needed to investigate the underlying mechanisms and to confirm any causal relationship between the change in underweight status and the risk of hip fracture.

## 5. Conclusions

We found out that the change in underweight status was strongly related to the risk of hip fracture in Korean adults over 40 years old. Adults who became underweight from being non-underweight had the highest risk of hip fractures, followed by those who were consistently underweight.

## Figures and Tables

**Table 1:** Baseline characteristics according to changes in underweight status.

Variables	CN	BN	BU	CU	*p*-Value
	(*n* = 1,666,364)	(*n* = 12,677)	(*n* = 13,141)	(*n* = 21,043)	
Age, years	55.2 ± 9.7	55.1 ± 11.4	57.7 ± 11.9	56.1 ± 11.8	<0.001
≥65 years, *n* (%)	300,388 (18.0)	2757 (21.8)	3855 (29.3)	5315 (25.3)	<0.001
Sex, *n* (%)					<0.001
Male	876,672 (52.6)	5604 (44.2)	5726 (43.6)	9861 (46.9)	
Female	789,692 (47.4)	7073 (55.8)	7415 (56.4)	11,182 (53.1)	
Height, cm	162.2 ± 8.7	161.0 ± 8.6	160.8 ± 8.5	161.7 ± 8.3	<0.001
Weight, kg	63.7 ± 10.3	50.7 ± 6.3	46.4 ± 5.2	45.6 ± 5.2	<0.001
BMI, kg/m^2^	24.2 ± 2.8	19.5 ± 1.5	17.9 ± 0.6	17.4 ± 0.8	<0.001
Smoking status, *n* (%)					<0.001
Non-smoker	1,040,628 (62.5)	8341(65.8)	8818 (67.0)	13,458 (64.0)	
Ex-smoker	311,799 (18.7)	1634(12.9)	1399 (10.7)	2304 (11.0)	
Current smoker	313,937 (18.8)	2702(21.3)	2924 (22.3)	5281 (25.0)	
Alcohol consumption, *n* (%)					<0.001
Non-drinker	953,558 (57.2)	8231 (64.9)	8837 (67.2)	13,933 (66.2)	
Moderate drinker	605,840 (36.4)	3887 (30.7)	3675 (28.0)	6176 (29.4)	
Heavy drinker	106,966 (6.4)	559 (4.4)	629 (4.8)	934 (4.4)	
Regular exercise, *n* (%)	374,781 (22.5)	2031 (16.0)	2222 (16.9)	3210 (15.3)	<0.001
Low income, *n* (%)	318,842 (19.1)	2668 (21.1)	2744 (20.9)	4274 (20.3)	<0.001
Comorbidities, *n* (%)					
Diabetes	202,779 (12.2)	761 (6.0)	1130 (8.6)	1195 (5.7)	<0.001
Hypertension	602,227 (36.1)	2510 (19.8)	3145 (23.9)	3725 (17.7)	<0.001
Dyslipidemia	427,031 (25.6)	1737 (13.7)	1746 (13.3)	2251 (10.7)	<0.001
CKD	100,980 (6.1)	617 (4.87)	817 (6.2)	1011 (4.8)	<0.001
Hip fracture, *n* (%)	6885 (0.41)	110 (0.9)	189 (1.4)	252 (1.2)	<0.001

CN, consistent non-underweight; BN, became non-underweight; BU, became underweight; CU, consistent underweight; CKD, chronic kidney disease.

**Table 2:** Hazard ratios for hip fracture according to changes in underweight status in adults ≥40 years old.

Group	Fracture Event (*n*)	Total FU Duration (PY)	IR (per 1000 PY)	Hazard Ratio (95% CI)			
				Model 1	Model 2	Model 3	Model 4
CN	6885	10,541,888.6	0.65	1	1	1	1
BN	110	78,972.9	1.39	2.14 (1.77–2.58)	1.63 (1.35–1.97)	1.57 (1.30–1.90)	1.72 (1.42–2.07)
BU	189	80,029.1	2.36	3.64 (3.15–4.21)	2.12 (1.83–2.45)	2.02 (1.75–2.34)	2.22 (1.96–2.53)
CU	252	129,072.9	1.95	3.01 (2.65–3.41)	2.09 (1.84–2.37)	1.98 (1.75–2.25)	2.18 (1.89–2.53)

FU, follow-up; PY, person–years; IR, incidence rate; CI, confidence interval; CN, consistent non-underweight; BN, became non-underweight; BU, became underweight; CU, consistent underweight. Incidence rate = fracture event/total follow-up duration. Model 1: non-adjusted. Model 2: adjusted for age and sex. Model 3: adjusted for age, sex, smoking, alcohol consumption, household income, and regular exercise. Model 4: adjusted for age, sex, smoking, alcohol consumption, household income, regular exercise, and comorbidities.

**Table 3:** Results of the subgroup analysis of hip fracture risk according to age and sex.

Category		Group	Hazard Ratio (95% CI)	*p*-Value	*p* for Interaction
Age	≥65 years	CN	1	<0.001	0.012
		BN	1.74 (1.12–2.71)		
		BU	3.13 (2.26–4.31)		
		CU	3.08 (2.37–4.01)		
	<65 years	CN	1	<0.001	
		BN	1.70 (1.38–2.09)		
		BU	2.02 (1.72–2.38)		
		CU	2.04 (1.77–2.36)		
Sex	Male	CN	1	<0.001	<0.001
		BN	1.95 (1.48–2.56)		
		BU	2.84 (2.32–3.49)		
		CU	2.83 (2.39–3.36)		
	Female	CN	1	<0.001	
		BN	1.55 (1.19–2.02)		
		BU	1.75 (1.42–2.15)		
		CU	1.73 (1.43–2.10)		

CI, confidence interval; CN, consistent non-underweight; BN, became non-underweight; BU, became underweight; CU, consistent underweight. Adjusted for age, sex, smoking, alcohol consumption, household income, regular exercise, and comorbidities.

## Data Availability

In accordance with Korean law, the study authors are not permitted to transfer any data files to a third party. However, data are available from the Korea National Health Insurance Sharing Service Institutional Data Access/Ethics Committee (https://nhiss.nhis.or.kr/bd/ay/bdaya001iv.do (accessed on 11 November 2021)) for researchers who meet the criteria for access to confidential data. The authors had no special access privileges to the data and other researchers will be able to access the data in the same manner as the authors.
